# From hPSCs to MSCs: differentiation strategies, pathways, and the emergence of common regulatory networks

**DOI:** 10.1186/s11658-026-00886-z

**Published:** 2026-02-24

**Authors:** Shengxian Liang, Zhuang Qian, Yichen Wang, Jingjing Huangfu, Wenjie Ren

**Affiliations:** 1 Clinical Medical Center of Tissue Engineering and Regeneration, Institutes of Health Central Plain, Henan Medical University, Xinxiang, 453003 China; 2 The First Affiliated Hospital, Henan Medical University, Xinxiang, 453199 China; 3 Henan Medical Key Laboratory for Research of Trauma and Orthopedics, The Third Affiliated Hospital, Henan Medical University, Xinxiang, 453003 China

**Keywords:** Human pluripotent stem cells, Mesenchymal stem/stromal cells, Differentiation protocols, Molecular mechanisms, Transcriptomic analysis, Regulatory networks

## Abstract

Mesenchymal stem/stromal cells (MSCs) derived from human pluripotent stem cells (hPSCs) represent a scalable and homogeneous source for regenerative medicine. To date, multiple differentiation protocols have been developed to direct hPSCs toward an MSC fate, with intermediate cell states arising from diverse lineages, including trophoblast, neural crest, mesoderm, and endoderm. Despite these divergent differentiation strategies, the induced MSCs exhibit similar phenotypes and biological functions, suggesting convergent molecular programs underlying MSC specification. In this review, we discuss current strategies for differentiating hPSCs into MSCs and summarize the key signaling pathways, with a focus on the transcriptional regulators that govern these lineage-specific differentiation routes. To identify common regulatory nodes across different lineages, we analyzed publicly available transcriptomic datasets from representative hPSC-to-MSC protocols deposited in the Gene Expression Omnibus (GEO) database. Comparative analysis revealed a core set of consistently dysregulated genes and enriched pathways, particularly those involved in extracellular matrix (ECM)-receptor interaction, focal adhesion, and the PI3K–Akt signaling pathway. Notably, SMAD3, along with AP-1 family members (JUN, JUND, FOSL1, FOSL2) and the associated regulatory targets (FN1 and COL1A1) emerged as recurrent hubs in mesenchymal commitment. These findings highlight both the plasticity and convergence in the induction of MSCs from hPSCs and provide a molecular framework for optimizing differentiation strategies and ensuring product consistency in regenerative applications.

**Graphical Abstract:**

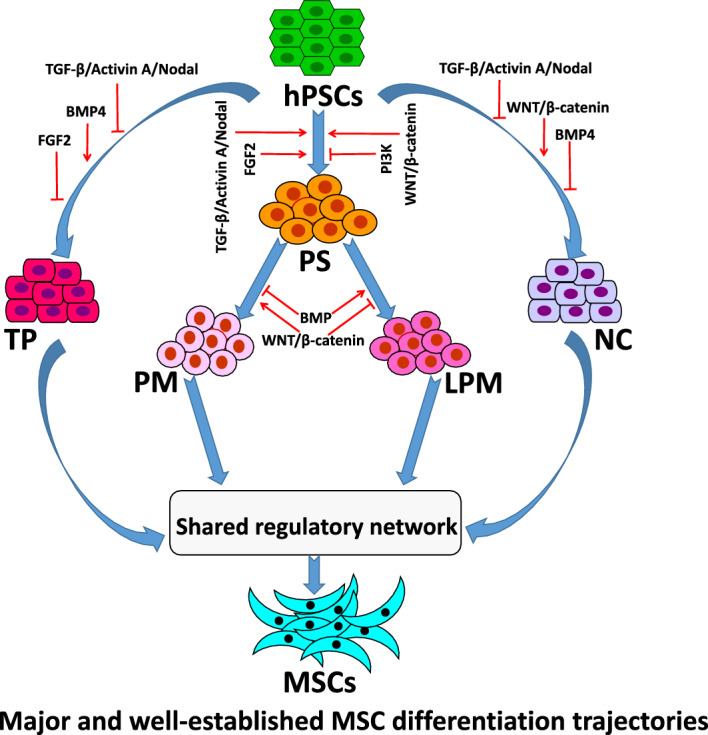

**Supplementary Information:**

The online version contains supplementary material available at 10.1186/s11658-026-00886-z.

## Introduction

Mesenchymal stem/stromal cells (MSCs) have emerged as a promising cell type for various therapeutic applications [1], primarily owing to their multiple lineage differentiation potential, immunomodulatory properties, and ease of isolation and expansion. These cells can be isolated from multiple tissues, including bone marrow, adipose tissue, umbilical cord blood, and placenta. However, owing to limitations including limited availability, donor-related variability, and risk of pathogen transmission, the large-scale clinical application of tissue-derived MSCs remains challenging. This has spurred extensive research into alternative methods for MSC generation.

Human pluripotent stem cells (hPSCs), including human embryonic stem cells (hESCs) and induced pluripotent stem cells (iPSCs), represent a promising unlimited source of MSCs [2, 3]. hPSCs have the ability to self-renew indefinitely and differentiate into cells of all three germ layers, making them an attractive starting material for generating lineage specific cells, including MSCs [2, 3]. Differentiating hPSCs into MSCs offers several advantages over MSCs derived from tissue sources. Firstly, it enables the generation of highly consistent and standardized cell populations, thereby minimizing batch-to-batch variability. Secondly, it circumvents the ethical concerns associated with the use of fetal tissue and overcomes the limited availability of adult stem cells. Finally, the genetic tractability of hPSCs enables the derivation of MSCs with enhanced therapeutic functions or precise genetic modifications, paving the way for improved cell-based therapies.

Since 2004, when Xu et al. [4] first derived the MSC-like cells from hESCs, numerous protocols for generating MSCs from hPSCs have been emerging including mushrooms after rain [5]. Similar to the broad distribution of MSCs within the human body, it appears that MSCs can be derived from multiple germ layers of hPSCs, including ectoderm, mesoderm, and endoderm [5–7]. In addition to differentiation from three germ layers, MSCs can also be derived via extraembryonic trophoblast, as well as embryoid bodies (EBs) that do not specify any germ layers [3, 5]. However, despite this diversity in developmental origins and induction strategies, the resulting hPSC-derived MSCs consistently exhibit a characteristic phenotype, including the fibroblast-like morphology, the surface marker expression pattern (CD44^+^/CD73^+^/CD90^+^/CD105^+^/CD34^−^/CD45^−^), the multilineage differentiation potential (osteogenic, chondrogenic, adipogenic), and the immunomodulatory functions [5]. This striking phenotypic and functional convergence suggests that diverse differentiation routes may ultimately activate a shared molecular program driving mesenchymal fate specification, although the nature of this program remains unknown.

This review summarizes recent advances in the differentiation of MSCs from hPSCs, with a focus on lineage-specific induction strategies and the underlying molecular mechanisms. Current approaches for directing hPSCs toward the mesenchymal lineage are discussed, with particular emphasis on key signaling pathways and transcriptional regulators that govern MSC specification. By dissecting the plasticity of differentiation trajectories and the core regulatory networks leading to MSCs, this review provides a framework for refining differentiation protocols and improving the consistency and quality of hPSC-derived MSCs.

## Current lineage differentiation strategies

### Trophoblast lineage

**Fig. 1:**
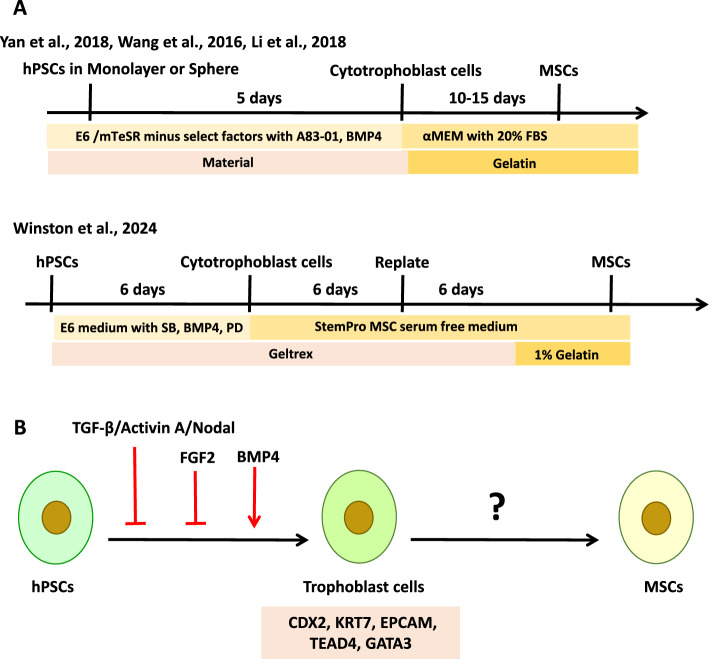
Derivation of MSCs from hPSCs via a trophoblast-like intermediate lineage. **A** Schematic representation of the stepwise differentiation protocols. **B** Key signaling pathways regulating each stage of the differentiation process. *SB* SB431542, *PD* PD173074. Graphic software: Microsoft PowerPoint 2013

The trophoblast lineage represents the first cell fate decision during early mammalian embryogenesis, emerging as the trophectoderm from the outer cells of the morula during the morula-to-blastocyst transition. These extraembryonic epithelial cells do not contribute to the embryo proper but instead give rise to the fetal component of the placenta and are essential for embryo implantation and placental development. It is well-established that the placenta is one of the major sources of MSCs. Given this knowledge, Wang et al. proposed the hypothesis that trophoblast cells might also serve as precursors of MSCs [3]. To obtain trophoblast cells, they treated the hPSCs with a combination of BMP4 and A83-01 for 5 days (Fig. 1A). BMP4 activated the BMP signaling pathway, while A83-01 inhibited the TGF-β–Activin–Nodal signaling pathways. After a 5-day induction with BMP4 and A83-01, trophoblast-like cells were dissociated and replated in MSC growth medium (containing FBS) without additional differentiation inducers. Following 14 days of culture, these cells exhibited characteristic MSC surface marker expression and demonstrated differentiation potentials into adipogenic, osteogenic, and chondrogenic lineages. Xu’s group further expanded this differentiation strategy under serum-free conditions using commercial medium [8] and in a three-dimensional (3D) culture system [9] (Fig. 1A). Although the characteristics of MSCs induced by these three methods are similar, the serum-free condition requires a longer differentiation time [8], while the three-dimensional (3D) condition significantly improves the differentiation efficiency [9]. These findings highlight the potential of 3D culture systems to enhance the efficiency of MSC differentiation, offering a promising approach for scalable and robust tissue engineering applications [9].

However, despite these significant methodological advances and robust phenotypic evidence of successful MSC differentiation in the studies mentioned above, the intermediate trophoblast-like cells were not adequately characterized. Wang et al. did not provide data to validate the identity of the induced trophoblast-like cells [3]. Similarly, Li et al. and Yan et al. relied solely on TROP2 expression as a marker to confirm the trophoblast identity [8, 9], which is insufficient given the complexity and heterogeneity of the trophoblast lineage. The lack of rigorous characterization of the intermediate trophoblast-like cells raises important questions about the authenticity and homogeneity of the starting population in these differentiation protocols. Given that BMP4 can induce not only trophoblast but also primitive endoderm and mesodermal fates under different conditions, and that A83-01-mediated TGF-β inhibition is also related to the ectoderm lineage specification, the precise identity of the “trophoblast-like” cells remains ambiguous without a comprehensive molecular signature. In general, validation should include a panel of lineage-specific markers at both mRNA and protein levels, including GATA3, CDX2, KRT7, etc. for trophoblast identity, and exclusion of markers associated with pluripotency (OCT4, NANOG) and three germ layers (e.g., SOX17 for endoderm, T for mesoderm, PAX6 for ectoderm). Furthermore, functional assays such as hormone secretion (e.g. HCG, progesterone) could provide additional evidence of trophoblast maturation. Without such validation, it remains uncertain whether the derived MSCs originate directly from a bona fide trophoblast lineage or from a heterogeneous or partially reprogrammed cell population.

Recently, Winston et al. [10] began to address this critical gap by applying transcriptome-wide profiling and immunostaining to characterize the intermediate trophoblast-like cells prior to MSC derivation. Their data demonstrated a robust upregulation of core trophoblast markers (including CDX2, KRT7, EPCAM, TEAD4, and GATA3). To improve the expression of trophoblast markers, Winston et al. [10] applied BMP4, SB431542, and low concentration of PD173074 (FGF2 inhibitor) to direct hPSCs toward the trophoblast lineage (Fig. 1A). After 6 days of induction, the cytotrophoblast cells were then replated into the MSC serum-free medium for further differentiation into MSCs.

Collectively, these studies demonstrate the phenotypic potential of hPSC-derived intermediates to give rise to MSCs via a trophoblast-like state. Previous research has established many protocols for directing both naive and primed hPSCs toward trophoblast lineage commitment in vitro [10, 11]. Notably, simultaneous inhibition of the SMAD2/3 (via TGF-β–Activin–Nodal blockade) and MEK1/2 (via FGF–ERK pathway inhibition) pathways, in combination with BMP4 signaling activation, has been shown to promote unidirectional differentiation of hPSCs toward a stable trophoblast fate [11]. However, the molecular mechanisms underlying the transition from trophoblast-like cells to MSCs remain largely unknown (Fig. 1B). Elucidating these mechanisms is critical to achieving robust and reproducible MSCs derivation.

### Neural crest lineage

**Fig. 2:**
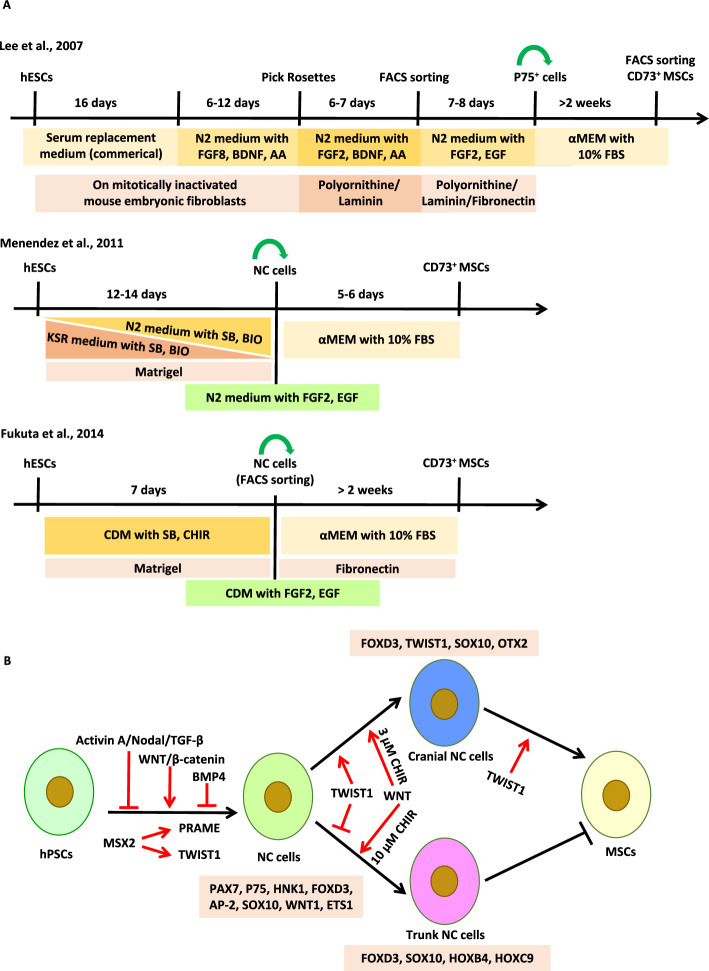
Derivation of MSCs from hPSCs via a neural crest intermediate lineage. **A** Schematic representation of the stepwise differentiation protocols. **B** Key signaling pathways regulating each stage of the differentiation process. *AA* ascorbic acid, *SB* SB431542, *BIO* (2’Z,3’E)-6-bromoindirubin-3’-oxime, *CHIR* CHIR99021, *CDM* chemically defined medium, the specific composition of CDM is detailed in the reference [19]. Graphic software: Microsoft PowerPoint 2013

Ectoderm is one of the three primary germ layers formed during embryogenesis, giving rise to two major domains: the surface ectoderm and the neural ectoderm. Neural crest (NC) cells are a group of transient, multipotent cells that emerge from the neural ectoderm [12]. These cells exhibit extensive migratory capacity and multipotency, contributing to various tissues and organs throughout the developing embryo [12]. Using green fluorescent protein (GFP) knock-in and Cre-recombinase mediated lineage tracking assays, Takashima et al. identified that the earliest wave of MSCs in the embryonic trunk is generated from SOX1^+^ neuroepithelium, partly through a NC intermediate stage [13]. Concurrently, Lee et al. reported the isolation, propagation, and directed differentiation of NC cells from hESCs [14]. To investigate the differentiation potential of NC cells toward mesenchymal lineages, Lee et al. cultured the hES-derived NC cells (p75^+^/HNK1^+^) in fetal bovine serum (FBS)-based MSC medium for at least 2 weeks (Fig. 2A). CD73^+^ cells were subsequently characterized by typical MSC surface markers and trilineage differentiation capacity. However, the strategy employed by Lee et al. to obtain NC cells is time-consuming and inefficient, thus severely limiting their utility in scale-up applications.

It is well-established that inhibition of BMP and Activin–Nodal–TGF-β signaling efficiently drives hESC differentiation toward neural progenitor cells. In addition, in vivo studies have demonstrated the critical role of WNT signaling in neural crest (NC) development [12, 15–17]. Based on this knowledge, Menendez et al. developed a highly efficient, one-step protocol to derive NC cells from hPSCs by activating WNT while inhibiting Activin–Nodal–TGF-β signaling pathways, which takes only 11 days and does not require fluorescence-activated cell sorting (FACS) sorting [18]. Menendez et al. further investigated the role of BMP signaling in NC induction [18], and found that the addition of Noggin, a BMP antagonist, had no significant effect on the efficiency of NC induction. In contrast, the addition of BMP4 markedly reduced the HNK1^+^P75^+^ NC cell population, indicating that BMP signaling antagonizes NC induction from hPSCs. The derived NC cells by Menendez et al. could differentiate into MSCs in FBS-based culture medium (Fig. 2A). Based on the precise signaling regulation strategy from Menendez et al., researchers subsequently developed several defined or xeno-free differentiation protocols to derive MSCs from hPSCs via NC stage [19, 20]. However, as is known, NC cells could give rise to connective tissue, neurons, glia, melanocytes, as well as mesenchymal derivatives. The aforementioned assays all derived MSCs from NC cells using a somewhat spontaneous differentiation method, merely switching NC culture medium to MSC culture medium without any specific inducers. Although the mechanisms underlying the differentiation of hPSCs into NC cells are relatively well-understood [18], which are mainly regulated by WNT activation and TGF-β inhibition, the process by which NC cells differentiate into MSCs remains unclear.

Understanding the development and lineage segregation of NC during mammalian embryogenesis may provide valuable insights into the signaling pathways that direct the NC cells toward differentiation into MSCs. Based on the anterior–posterior axis, NC cells can be grouped into cranial, cardiac, vagal, trunk, and sacral populations [21]. Cranial NC cells, which originate from the dorsal neuroepithelium before neural tube closure, exhibit unparalleled mesenchymal plasticity compared with their trunk counterparts [21]. They contribute to a wide variety of mesenchymal structures in the head, including bone, cartilage, and connective tissues, and have been identified as a major source of MSCs [21–24]. Therefore, activating key signaling pathways involved in cranial NC formation and its further specification may improve the derivation of MSCs from NC cells.

The specification and differentiation of cranial NC cells are governed by a cohort of transcription factors with specialized roles in this unique embryonic population. Among these, FOXD3, TWIST1, and SOX10 emerge as pivotal regulators that orchestrate cranial NC development through distinct yet interconnected mechanisms:

FOXD3, one of the earliest molecular markers of NC, is indispensable for the maintenance of the multipotent NC cells. In animal studies, highly conserved NC1 and NC2 are two enhancers of *FoxD3* that mediate its expression in cranial and trunk NC lineages, respectively [25]. Transcription factors* Pax7*, *Msx1*/2 and *Ets1* are critical for activating NC1 regulatory element to promote the expression of *Foxd3* in cranial NC cells [25]. Tissue-specific deletion of *Foxd3* in mice results in a severe loss of NC-derived tissues [26]. Furthermore, *Foxd3* mediates a fate restriction decision in NC cells. In the cranial region, loss of *Foxd3* leads to premature differentiation toward osteogenic and chondrogenic fates in mice, indicating a bias towards mesenchymal lineage [27].

TWIST1, a basic helix-loop-helix (bHLH) transcription factor, is specifically activated in cranial NC cells [24]. *Twist1*-knockout mouse embryos display defective ectomesenchyme development, significantly impair skeletogenic differentiation, and result in a reduction of mesenchymal derivatives and an increase in neural fates [24, 28]. Interestingly, sustained ectopic expression of *Twist1* could reverse the trunk NC cells to a mesenchymal fate at the expense of neuronal sensory, autonomic, and glial fates [24]. The specific activation of *Twist1* in cranial NC cells and the effects of its mutation and ectopic expression highlight its importance in orchestrating the complex processes of NC cell differentiation and fate decisions. In human cells, Zhang et al. confirmed the crucial roles of *TWIST1* in MSC induction from hESCs via the NC lineage [29]. Furthermore, they identified MSX2 as an upstream regulator of *TWIST1* [29]. However, inconsistent with the results obtained from mouse and zebrafish embryos that *Twist1* deficiency led to persistent expression of *Sox10* [28, 30], Zhang et al. found that *TWIST1* knockdown in hESCs significantly repressed *SOX10* expression. The discrepancy may be due to the distinct functions of *TWIST1* and *SOX10* at the early stages of NC development and the later stages of NC differentiation.

SOX10, another specific and early marker of NC cells, is one of the key specifiers that regulate multiple stages of NC development, including proliferation, migration, and differentiation. Highly conserved SOX10E2 and SOX10E1 are two enhancers of SOX10 that mediate its expression in cranial and trunk NC lineages, respectively [31]. Transcription factors *Sox9*, *Ets1*, and *c-Myb* are critical for activating SOX10E2 to promote the expression of *Sox10* in cranial NC cells [31]. Consistent with its essential role, knockout or knockdown of *SOX10* in human iPSCs or ESCs significantly reduces the NC population and impairs the generation of mesenchymal cells, as well as neuronal and glial differentiation [29, 32]. Previous studies indicate that SOX10 expression is tightly regulated by transforming growth factor-beta (TGF-β) signaling, while the functional consequences of this regulation are highly stage-dependent. During NC induction from hPSCs, TGF-β signaling must be inhibited to allow proper specification of SOX10^+^ NC cells. This principle is exemplified by the widely used protocol of Menendez et al., which combines CHIR99021 (WNT activator) with SB431542 (TGF-β inhibitor) to efficiently generate NC cells [18]. In contrast, during the subsequent NC-to-MSC transition, TGF-β signaling plays a distinct role, its activation promotes mesenchymal commitment. Suppression of Sox10 in mouse NC cells induces a fate switch from neural to mesenchymal fate through TGF-β signaling [33], indicating that the downregulation of SOX10 mediated by TGF-β is permissive for mesenchymal differentiation at this later stage. In addition, WNT signaling further modulates this dynamic regulation. In *Xenopus*, WNT activation enhances Sox10^+^ NC formation, whereas WNT inhibition suppresses Sox10 expression in the NC-forming region [34], indicating its positive role in early NC induction. In human cells, level of WNT/β-catenin activity also determines NC axial identity (Fig. 2B): low doses of WNT (3 μM CHIR99021) lead to anterior OTX2^+^/HOX^−^ NC (cranial), while high doses (10 μM CHIR99021) result in posterior OTX2^−^/HOX^+^ NC (trunk) with poor capacity to generate mesenchymal lineages [35]. Moreover, WNT/β-catenin signaling remains essential beyond the NC induction phase. Choe et al. found that the WNT/β-catenin signaling pathway is required for the transition of the mouse NC cells to MSCs [22], indicating that sustained WNT activity supports mesenchymal commitment even after NC specification. Taken together, current evidence supports a model in which TGF-β and WNT signaling play stage-specific roles during NC development: their inhibition and activation, respectively, are required for NC induction, while their subsequent activation may facilitate mesenchymal commitment. Although direct evidence linking dual TGF-β/WNT activation to enhanced NC-to-MSC transition remains limited, this hypothesis warrants experimental testing in human in vitro models.

### Mesoderm lineage

Among the three germ layers, mesodermal cells give rise to the majority of adipose, skeletal, and connective tissues in vertebrates. As these tissues serve as the primary sources for isolating tissue-resident MSCs in adults, mesoderm is recognized as the dominant embryonic origin of MSCs [36]. During embryonic development, the mesoderm lineages initially originate from the epiblast cells, and subsequently specialize into four regions including the axial mesoderm (giving rise to the notochord), the lateral plate mesoderm (LPM), the intermediate mesoderm (IM), and the paraxial mesoderm (PM) [36, 37]. Among these mesodermal derivatives, the IM primarily contributes to the formation of the urogenital system, including the kidneys and gonads [37]. The PM plays a particularly important role in generating somites, which are transient structures that further differentiate into skeletal muscle, cartilage, dermis, and part of the axial skeleton [38]. The LPM, on the other hand, contributes to the body wall, limbs, the heart and the visceral organs [38]. Tissues derived from PM and LPM, such as bone, adipose tissue, and limb mesenchyme, are known to harbor populations of tissue-resident MSCs. This suggests that PM and LPM may serve as embryonic origins of MSCs [36].

**Fig. 3:**
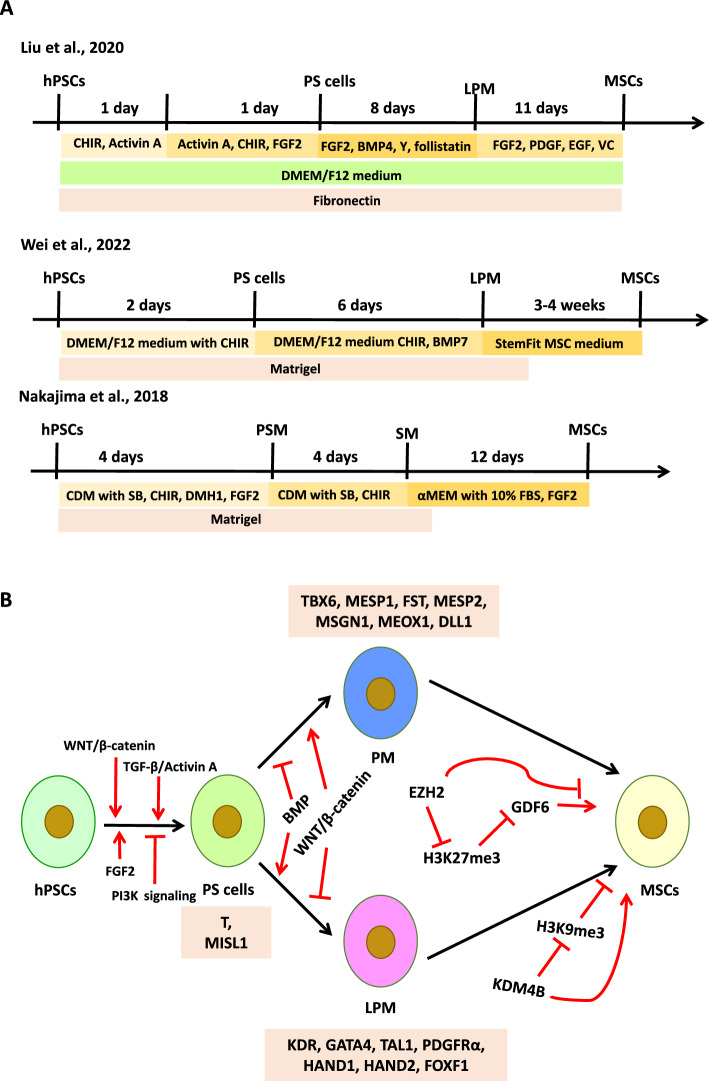
Derivation of MSCs from hPSCs via mesoderm intermediate lineage. **A** Schematic representation of the stepwise differentiation protocols. **B** Key signaling pathways regulating each stage of the differentiation process. *Y* Y-27632, *CHIR* CHIR99021, *VC* ascorbate, *SB* SB431542, *CDM* chemically defined medium, the specific composition of CDM is detailed in the reference [42]. Graphic software: Microsoft PowerPoint 2013

To identify the precursors that give rise to MSCs during mesoderm development, Vodyanik et al. applied an OP9-based coculture system to specify mesoderm from hESCs, and found that MSCs originate from a population of APLNR-positive cells with a LPM gene profile, including markers such as FOXF1 and HAND1 [7]. Consistent with their LPM origin [7], MSCs were also isolated as a by-product during cardiomyocyte differentiation of hPSCs. Employing the EB-based differentiation strategy, Wei et al. demonstrated that supplementation of the culture medium with a p38 MAPK inhibitor (SB203580) promoted cardiomyocyte specification, and MSCs emerged concomitantly alongside the beating cardiomyocytes after EBs were plated on gelatin-coated dishes [39]. More specifically, Wei et al. directed the differentiation of hPSCs toward the primitive streak (PS) stage and further specified them into LPM through staged modulation of the WNT and BMP signaling pathways, and these LPM cells were further differentiated into MSCs through spontaneous differentiation in StemFit MSC medium (Fig. 3A) [40]. Similarly, Liu et al. developed a chemically defined and stepwise protocol that fully recapitulates the major stages of MSC development in vitro, including the transition from hPSCs to PS and subsequently to LPM [41]. By introducing small molecules such as FGF2, PDGF, EGF, and ascorbic acid at defined stages (Fig. 3A), Liu et al. significantly enhanced both the differentiation efficiency and the osteogenic and chondrogenic potential of the derived MSCs [41]. Besides the LPM, PM which gives rise to the somites and further differentiates into the sclerotome and dermomyotome, has been identified as another source for MSC generation [42]. Using the TGF-β off/WNT-on/BMP-off/FGF-on signaling strategy, Nakajima et al. successfully induced somite from human iPSCs through a PM intermediate stage (Fig. 3A), and the induced somite could further differentiate into MSCs after 12 days of culture in medium supplemented with FBS and FGF2 [42].

The underlying molecular mechanisms that specify hPSCs to PM and LPM are governed by a complex interplay of signaling pathways and transcriptional regulators (Fig. 3B). It has been well-established that the induction of PS intermediates is a prerequisite for subsequent mesoderm patterning, which is primarily driven by the activation of WNT, TGF-β/Activin A, FGF, and inhibiting PI3K signaling pathway [38]. Upon PS formation, graded activation of WNT and BMP signaling directs lineage specification into PM and LPM. Specifically, for hPSC differentiation in vitro, exogenous BMP activation or WNT inhibition induces LPM formation and represses PM generation. In contrast, blocking the BMP signaling pathway or activating WNT favors PM formation and abrogates LPM development [38]. Moreover, key transcription factors including TBX6, NANOG, CDX2 were identified to be critical for the lineage diversification of mesoderm cells [43, 44]. Generally, researchers use the expression of *TBX6*, *MESP1*, *FST*, *MESP2*, *MSGN1*, *MEOX1*, and *DLL1* as PM markers, and *GATA4*, *TAL1*, *PDGFR*α, *KDR*, *HAND1*, *HAND2*, and *FOXF1* as LPM markers.

Although MSCs have been successfully derived from both PM and LPM through directed differentiation of hPSCs, the precise molecular mechanisms governing this lineage transition remain poorly understood. Recently, several studies have shown that modulating pathways or factors such as IKK/NF-κB, EZH2, and KDM4B can markedly improve the efficiency of MSC differentiation from mesoderm progenitors [45–47]. Deng et al. demonstrated that inhibition of the IKK/NF-κB signaling pathway enhances the differentiation efficiency of hESCs into MSCs, resulting in a three-fold increase in the proportion of MSCs (from 3.5 to 11.5%) compared with the control group [45]. Subsequently, researchers from the same laboratory performed an epigenome-wide analysis comparing hESCs and MSCs, and found that EZH2 expression was significantly downregulated during the transition of hESCs to MSCs. Notably, pharmacological inhibition of EZH2 markedly enhanced mesoderm specification, likely through a reduction in H3K27me3 levels. Furthermore, inhibition of EZH2 using the specific inhibitor GSK126, or its functional knockdown, selectively enhanced the differentiation of hESCs toward the MSC lineage. This effect was partially recapitulated by ectopic expression of GDF6, suggesting its involvement in the EZH2-regulated differentiation program [46, 47]. In another study, Liu et al. demonstrated that ascorbate is required for the specification of MSCs from LPM, not through its antioxidant function, but rather through the activity of ascorbate/iron-dependent dioxygenases, which enhance the expression of KDM4B (also known as JMJD2B), a JmjC domain-containing histone demethylase [41]. Studies by Liu et al. and Yu et al. suggested that reduction of histone methylation levels is beneficial for the efficient derivation of MSCs from hPSCs [41, 47]. Taken together, although these studies do not fully elucidate the complete molecular mechanisms underlying the differentiation of hESCs into MSCs via the mesodermal lineage, they provide valuable insights and important molecular clues that contribute to our understanding of this process.

### Endoderm lineage

Despite the fact that MSCs have been isolated from endoderm-derived organs such as lung and liver [48], there is limited knowledge regarding the potential of hPSCs to differentiate into MSCs via the endodermal lineage cells. In 2023, Zhang et al. reported that SOX17^+^ definitive endoderm progenitors could further differentiate into MSCs [6]. In this protocol, hESCs were first induced toward a mesoendoderm fate by treatment with CHIR99021 and Activin A for 1 day. Subsequently, definitive endoderm specification was achieved by an additional 2-day treatment with Activin A. The resulting definitive endoderm progenitors were then cultured in αMEM medium supplemented with FBS, FGF2, insulin, NEAA, and GlutaMAX for 12 days. Finally, MSC-like cells were obtained after 2–3 passages [6]. Short-term treatment of the definitive endoderm progenitors with CHIR99021 and SB431542, which activate Wnt signaling and inhibit Nodal/Activin A signaling, respectively, significantly promoted the expression of MSC markers. Interestingly, these small molecules also showed strong effects in directing hPSCs toward MSCs via intermediate lineages such as trophoblast, NC, and mesoderm, indicating a broader role of these two pathways in modulating lineage transitions during MSC induction.

In summary, a study by Zhang et al. has provided valuable insights into the endodermal origin of MSCs from hPSCs [6], while the current methodologies for generating MSCs through this lineage remain limited. Moreover, the precise molecular mechanisms underlying the transition of endoderm to MSCs are not fully elucidated. Given these challenges, there is an urgent need for further research to optimize the protocols, explore the underlying molecular targets, and develop more efficient and reproducible strategies for generating functional MSCs from hPSCs via the endodermal lineage.

## Regulatory networks and hub genes underlying hPSC differentiation into MSCs

**Fig. 4:**
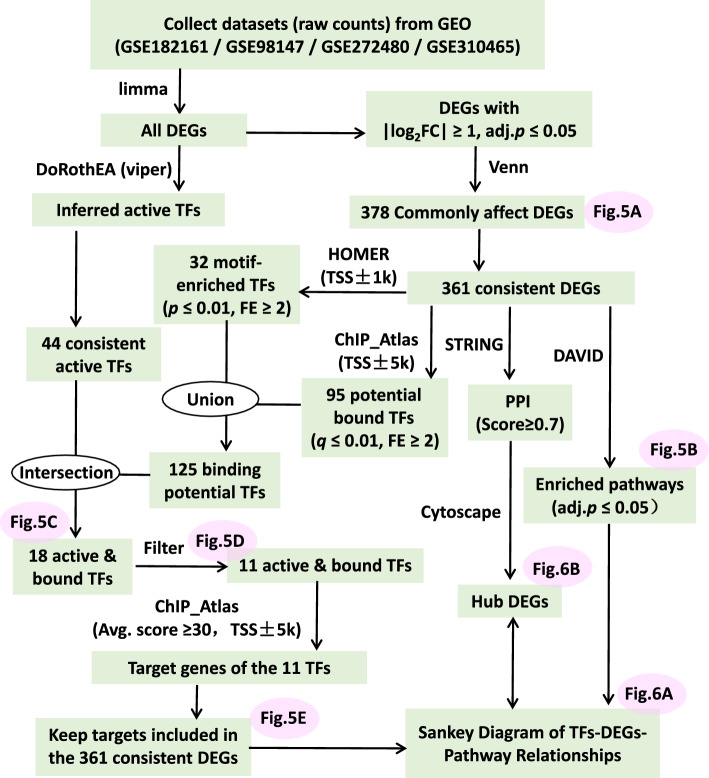
Flowcharts for the analyses of datasets collected from Gene Expression Omnibus (GEO). Graphic software: Microsoft PowerPoint 2013

**Fig. 5:**
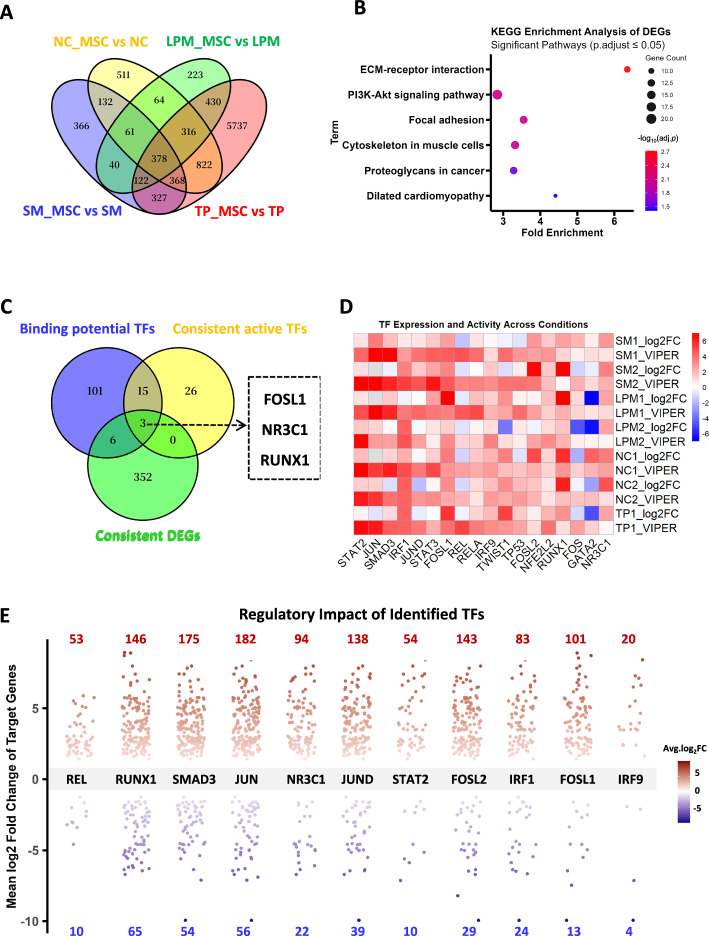
Integrated analysis of the four transcriptomic datasets from GEO. **A** Venn diagram analysis to identify the common differentially expressed genes (DEGs) (|log_2_FC|≥ 1) across the four datasets. **B** Kyoto Encyclopedia of Genes and Genomes (KEGG) pathway enrichment analysis of the 361 consistent DEGs. The six significantly (adj.*p* < 0.05) enriched pathways are shown. **C** Intersection of transcription factors identified by “HOMER or ChIP-Atlas” (Binding potential TFs), DoRothEA (Consistent active TFs), and the 361 consistent DEGs. **D** Heatmap of the 18 common transcription factors (TFs) identified in **C**. Columns represent TFs, rows represent groups, “SM1” = “GSE98147: SM_MSCs versus SM”, “SM2” = “GSE310465: SM_MSCs versus SM”, “LPM1” = “GSE310465: LPM_MSCs versus LPM”, “LPM2” = “GSE182161: LPM_MSCs versus LPM”, “NC1” = “GSE310465: NC_MSCs versus NC”, “NC2” = “GSE272480: NC_MSCs versus NC”, “TP1” = “GSE272480: TP_MSCs versus TP”. **E** The 11 potential TFs and their target genes which were identified among the 361 consistent DEGs. Graphic software: Panels **A** and **C** were generated with Venny 2.1(https://bioinfogp.cnb.csic.es/tools/venny/index.html), panels **B** and **E** with R/ggplot2, panel 5D with R/pheatmap

To identify conserved molecular signatures during hPSC differentiation into MSCs across germ layers, we integrated transcriptomic datasets from four studies (GSE310465, GSE272480, GSE182161, and GSE98147) deposited in GEO (https://www.ncbi.nlm.nih.gov/gds/), representing distinct developmental lineages including TP, NC, LPM, and PM (Supplementary Table S1). The analysis workflow is illustrated in Fig. 4. We specifically focused on the transition from lineage-specific progenitor cells to MSCs, rather than the initial differentiation from hPSCs, to minimize confounding effects associated with pluripotency and early lineage specification. Differentially expressed genes (DEGs) were identified in each dataset by comparing these intermediate progenitor states, including TP, NC, LPM, and somite (SM, derived from PM), to their respective MSC derivatives, using limma (Fig. 4). Venn analysis revealed a total of 378 common DEGs across all four lineages (Fig. 5A), of which 361 exhibited consistent expression trends (i.e., uniformly up- or downregulated), suggesting a conserved transcriptional program during mesenchymal commitment independent of germ layer origin.

To further characterize the functional roles of these 361 consistent DEGs, we conducted KEGG pathway enrichment analysis. A total of six pathways were significantly enriched (adj. *p* < 0.05), five of which may be strongly associated with mesenchymal biology (Fig. 5B). These included focal adhesion and ECM-receptor interaction (cell–matrix adhesion), PI3K–Akt signaling pathway (cell survival, migration, and differentiation), cytoskeleton in muscle cells (cell morphology), and Proteoglycans in cancer (cell survival, migration). Notably, the ECM-receptor interaction term emerged as the most statistically significant, exhibiting the highest fold enrichment value, suggesting a critical role for ECM-mediated signaling in directing MSC generation.

Given that coordinated gene expression programs are orchestrated by specific transcription factors (TFs), we sought to identify master regulators underlying the observed transcriptomic changes. First, we analyzed the regulatory regions of the 361 consistent DEGs using ChIP-Atlas and HOMER, with defined genomic windows: ± 5 kb around transcription start sites (TSSs) for ChIP-Atlas (based on aggregated public ChIP-seq data), and ± 1 kb around TSSs for HOMER (used in de novo motif enrichment analysis). This combined analysis identified 125 TFs, with either ChIP-seq peak enrichment in ChIP-Atlas (*q* < 0.01, FE > 2) or significant motif enrichment in HOMER (*p* < 0.01, FE > 2) (Fig. 5C, Supplementary Table S2–S3). Second, we applied DoRothEA to infer functional TF activity from the full set of DEGs across all groups, which revealed 44 TFs exhibiting consistent activity, as quantified by VIPER scores (Fig. 5C, Supplementary Table S4). The intersection of these two independent TF sets yielded 18 high-confidence candidates (Fig. 5C). To further prioritize robust regulators, we selected TFs that either (i) showed consistent differential expression (|log_2_FC| > 1 and same sign) or (ii) displayed consistent regulatory activity (|VIPER score|> 1 and same sign) in all groups. Finally, this integrative filtering yielded 11 TFs, including SMAD3, IRF1, JUND, FOSL1, REL, IRF9, FOSL2, STAT2, JUN, RUNX1, and NR3C1 (Fig. 5D). For these 11 TFs, we extracted their experimentally supported target genes from ChIP-Atlas, and identified the subset overlapping with our 361 consistent DEGs (Fig. 5E). Among them, JUN, SMAD3, and RUNX1 exhibited the broadest regulatory influence, targeting 238, 229, and 211 DEGs, respectively, implicating their potential roles as central hubs in the transcriptional program. In contrast, IRF9 regulated the fewest DEGs (*n* = 24), suggesting a more limited regulatory scope within the network.

**Fig. 6:**
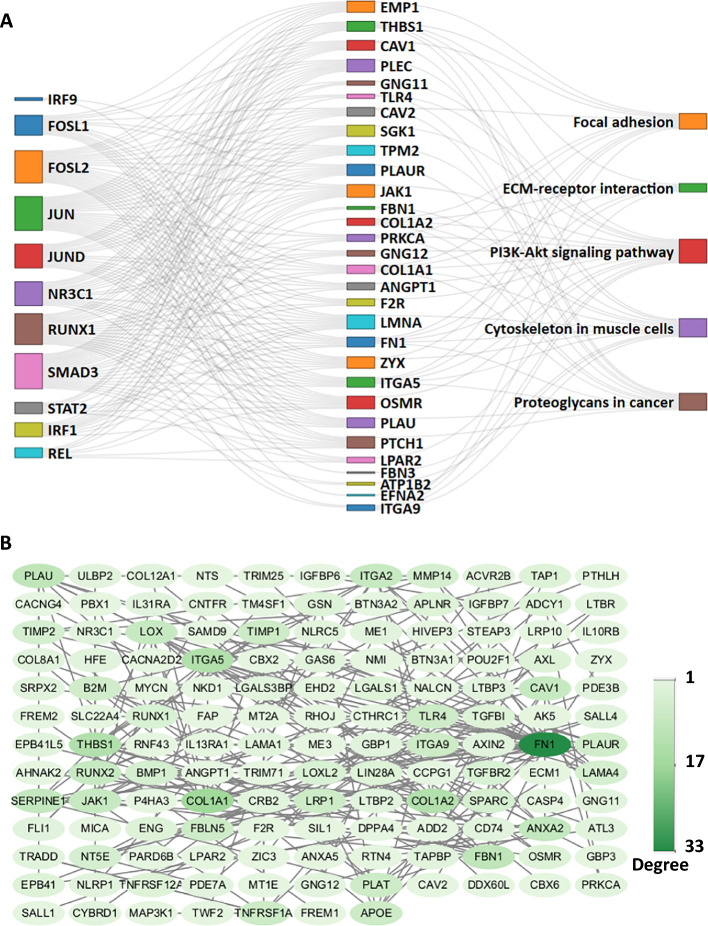
Integrated TF–target–pathway and protein–protein interactions (PPI) network analysis. **A** Sankey diagram showing the relationships between TFs, KEGG pathways and their common target DEGs. **B** PPI network of the 361 consistent DEGs. Nodes represent proteins; edges represent known physical or functional interactions curated from STRING with confidence of 0.7. Graphic software: Panel **A** with R/networkD3, panel **B** with Cytoscape v3.10.3

To visualize the regulatory and functional relationships among the 11 TFs and the five KEGG pathways associated with mesenchymal biology, we constructed a Sankey diagram depicting the connections between TFs, shared target genes, and enriched pathways. As shown in Fig. 6A, the network topology is highly consistent with our earlier predictions. IRF9 exhibited the weakest association with KEGG pathways, forming only two connections (via JAK1 and FN1). IRF1, REL, and STAT2 also showed limited connectivity. Collectively, these four TFs are canonical mediators of innate immune signaling [49, 50]. Although trace cytokines may be present in serum-containing media, there is no evidence that they are required for mesenchymal lineage specification from pluripotent precursors. Thus, they are likely dispensable for fate determination, and their transcriptional activity probably reflects the acquisition of immunomodulatory competence in derived MSCs [51, 52].

In contrast, the other seven TFs, including JUN, JUND, FOSL1, FOSL2, SMAD3, NR3C1, and RUNX1 shared more DEGs with the enriched KEGG pathways. Among them, JUN, JUND, FOSL1, and FOSL2 all belong to the AP-1 family known to integrate diverse signals to control cell proliferation, differentiation, and fate decisions [53–55]. Notably, JUN and JUND can form homodimers or heterodimers with FOSL1/2 [53, 54], and all four AP-1 members exhibited consistently high inferred regulatory activities across all conditions (Fig. 5D), suggesting that they are functionally engaged in the MSC specification. This is further supported by their strong connectivity to all the five KEGG pathways in the Sankey network (Fig. 6A). In parallel, SMAD3, which is the canonical downstream effector of TGF-β signaling, also displayed high inferred regulatory activity and extensive connectivity across all five mesenchymal-related pathways (Figs. 5D, 6A). Consistent with this, Smad3-null mice display negative effects on the osteogenesis and adipogenesis [56, 57], underscoring a positive regulatory role for SMAD3 in mesenchymal lineage commitment. Critically, SMAD3 is known to physically and functionally cooperate with AP-1 family members at composite enhancer elements to coactivate gene expressions [58]. The concurrent high activity of both SMAD3 and AP-1 factors therefore suggests the existence of a synergistic transcriptional module that directs the MSC specification.

Interestingly, despite their substantial overlap of DEGs with KEGG pathways, and strong transcriptional upregulation, RUNX1 (average log_2_FC = 5.3; range: 1.7–9.0) and NR3C1 (average log_2_FC = 3.1; range: 2.4–4.7) displayed low inferred regulatory activity (Fig. 5D), suggesting their induction may reflect downstream or permissive responses rather than a primary driver. This is particularly relevant for RUNX1, whose role in MSC biology remains controversial: while some studies report it promotes osteo-chondrogenic differentiation [59, 60], others including Shah et al., show that RUNX1 loss enhances mesenchymal cell emergence, implying RUNX1 normally suppresses MSC specification during early patterning [61, 62]. The apparent contradiction may be explained by its isoform diversity and stage-specific functions. The RUNX1 gene produces multiple isoforms through alternative promoter usage, including the distal P1 promoter driving RUNX1c (transactivation capacity), and the proximal P2 promoter gives rise to RUNX1a/b (dominant-negative inhibitors) [60, 63]. Critically, functional genomics study revealed that a noncoding regulatory element at the RUNX1 locus represses the long isoform (RUNX1c) expression and osteogenesis, while the MSCs induction from iPSCs was not affected [62], suggesting its major role lies in later lineage maturation rather than early fate commitment. Therefore, the strong upregulation of RUNX1 expression coupled with low regulatory activity (Fig. 5D) may reflect predominant expression of nonactivating isoforms and/or cis-mediated repression of RUNX1c, representing an “unlocked but not activated” state and further underscoring that mRNA abundance alone is insufficient to infer regulatory potency.

To further explore the functional organization of the 361 consistent DEGs beyond transcriptional regulation, we constructed a protein–protein interaction (PPI) network using STRING database (interaction score > 0.7) and visualized in Cytoscape (version 3.10.3). As shown in Fig. 6B, FN1 and COL1A1 emerged as top hub genes based on degree centrality, reflecting their extensive connectivity within the interactome. This regulatory relationship is further reflected in the Sankey diagram of TF–gene–pathway relationships, where the two genes map to the five KEGG pathways, and most of the TFs with high VIPER scores, such as SMAD3 and JUN (Fig. 6A). FN1 and COL1A1 encode major extracellular matrix proteins of fibronectin and type I collagen, respectively, which serve as ligands for cell surface integrins, and have been identified as targets of TGF-β signaling pathway [64, 65]. Notably, FN1 has been demonstrated to be involved in the lineage differentiations of ESCs, TP, mesoderm, and NC [55, 66–68], and this involvement has been attributed to SMAD3 or JUN in distinct differentiation models [55, 67]. Moreover, the in vivo experiment with mice have indicated a positive role of TGF-β signaling pathway in the generation of MSCs from NC [33]. Together, these findings support a hypothesis that SMAD3 and AP-1 family members with consistently high inferred activity during mesenchymal commitment may function as a cooperative regulatory module that orchestrates the expression of hub genes including FN1 and COL1A1, thereby facilitating the MSC specification from early germ layer progenitors.

## Concluding remarks

Over the past two decades, a remarkable diversity of differentiation protocols has emerged, guiding hPSCs toward the mesenchymal lineage through distinct developmental trajectories, including ectodermal, mesodermal, endodermal, and even extraembryonic pathways. This plasticity in differentiation strategies highlights the robustness and flexibility of hPSCs in acquiring a mesenchymal fate. However, mechanistic insights into the lineage-specific specification of hPSC-derived intermediate progenitor cells toward MSCs remain scarce. Most current protocols rely on spontaneous differentiation in MSC medium, lacking precise temporal control or defined molecular cues. This leads to poor reproducibility across laboratories and variable differentiation outcomes, further worsened by reagent batch variability, including fetal bovine serum used during MSC expansion.

We analyzed the common DEGs from omics datasets of MSC induction across distinct germ layers. By integrating TF prediction, KEGG pathway enrichment, and PPI network analysis, we identified several key molecules that may regulate the commitment to MSC fate. Functional studies are needed to confirm the roles of these candidate molecules in MSC specification. Moreover, emerging technologies such as single-cell RNA sequencing offer powerful tools to dissect how precursor cells from distinct germ layers can converge on the MSC phenotype even in the absence of specific inductive signals, rather than differentiating into other lineage-specific somatic cell types. Understanding the core regulatory network driving this convergent differentiation may ultimately enable the rational engineering of MSCs with enhanced lineage fidelity and therapeutic function.

## Supplementary Information

Supplementary material 1.

## Data Availability

The datasets analyzed during the current study are available in the GEO DataSets (https://www.ncbi.nlm.nih.gov/gds/).

## Abbreviations

MSCs: Mesenchymal stem cells
hPSCs: Human pluripotent stem cells
iPSCs: Induced pluripotent stem cells
hESCs: Human embryonic stem cells
TP: Trophoblast
NC: Neural crest
LPM: Lateral plate mesoderm
PM: Paraxial mesoderm
TF: Transcription factors
DEG: Differentially expressed genes
PPI: Protein–protein interaction
PS: Primitive streak
3D: Three-dimensional
IM: Intermediate mesoderm
