# Supplementary material for: From hPSCs to MSCs: differentiation strategies, pathways, and the emergence of common regulatory networks
Source: Cell Mol Biol Lett. 2026 Feb 24;31:43. doi: 10.1186/s11658-026-00886-z (PMC13040787; doi:10.1186/s11658-026-00886-z)
Supplement: Supplementary file 1 — Supplementary material 1. [file 11658_2026_886_MOESM1_ESM.docx]

**From hPSCs to MSCs: Differentiation Strategies, Pathways, and the Emergence of Common Regulatory Networks**

Shengxian Liang ^1,*^, Zhuang Qian ^1^, Yichen Wang ^2^, Jingjing Huangfu ^3^, Wenjie Ren ^1,2,3,*^

^1^ Institutes of Health Central Plain, Clinical Medical Center of Tissue Engineering and Regeneration, Henan Medical University, Xinxiang 453003, China.

^2^ The First Affiliated Hospital, Henan Medical University, Xinxiang 453199, China.

^3^ Henan Medical Key Laboratory for Research of Trauma and Orthopedics, The Third Affiliated Hospital, Henan Medical University, Xinxiang, China,453003

^*^ Corresponding author. E-mail: lsx@xxmu.edu.cn, 171001@xxmu.edu.cn

Table S1 Sample assignment

| Lineage | GEO Accession | Sample (GSM ID) | Reference |
| --- | --- | --- | --- |
| NC | GSE310465 | GSM9301769-70 for NC cells, GSM9301773-74 for MSCs derived from NC cells. | ^[1]^ |
|  | GSE272480 | GSM8403307-08 for NC cells, GSM8403300 for MSCs derived from NC cells. | ^[2]^ |
| LPM | GSE310465 | GSM9301787-88 for LPM cells, GSM9301791-92 for MSCs derived from LPM cells. | ^[1]^ |
|  | GSE182161 | GSM5519519-20 for LPM cells, GSM5519521-22 for MSCs derived from LPM cells. | ^[3]^ |
| PM | GSE310465 | GSM9301781-82 for SM cells (PM lineage), GSM9301785-86 for MSCs derived from SM cells. | ^[1]^ |
|  | GSE98147 | GSM2588538-40 for SM cells, GSM3266827-29 for MSCs derived from SM cells. | ^[4]^ |
| TP | GSE272480 | GSM8403303-04 for TP cells, GSM8403299 for MSCs derived from TP cells. | ^[2]^ |

Table S2 TFs (Log_10_ *q*-value≤-2, FE≥2) with ChIP-Atlas-supported binding in promoters (Experiment type: Chip_TFs and others, Cell type Class: All, Threshold for Significance: 100, Distance range from TSS: ±5 kb) of the 361 consistent DEGs.

| Feature | Log Q-val | FE | Feature | Log Q-val | FE | Feature | Log Q-val | FE |
| --- | --- | --- | --- | --- | --- | --- | --- | --- |
| ESR1 | -2.8 | 52.1 | IRF2 | -5.6 | 3.2 | RNF2 | -17.8 | 2.3 |
| NCOA3 | -2.1 | 19.5 | SMAD3 | -6.0 | 3.1 | SMARCA4 | -11.7 | 2.3 |
| GCM1 | -2.1 | 19.5 | JUN | -13.1 | 3.0 | NOTCH1 | -5.4 | 2.3 |
| MLLT1 | -3.2 | 15.3 | EPAS1 | -5.0 | 3.0 | SMAD2 | -2.3 | 2.3 |
| IRF3 | -6.6 | 12.7 | FOSL1 | -10.5 | 3.0 | DPF2 | -3.8 | 2.3 |
| SUPT6H | -2.1 | 7.4 | PRDM1 | -4.1 | 2.9 | FOSL2 | -9.8 | 2.3 |
| BRD4 | -2.5 | 5.9 | ZNF596 | -3.8 | 2.8 | MTF2 | -9.2 | 2.3 |
| MLLT3 | -2.8 | 5.8 | WWTR1 | -4.4 | 2.8 | FOXL2 | -2.7 | 2.3 |
| ERG | -3.0 | 4.8 | RELA | -7.4 | 2.8 | TEAD1 | -6.6 | 2.3 |
| GPS2 | -2.7 | 4.8 | JUND | -3.8 | 2.7 | JUNB | -13.3 | 2.2 |
| TRPS1 | -4.5 | 4.7 | SMARCE1 | -9.3 | 2.7 | TWIST1 | -3.0 | 2.2 |
| MED1 | -10.2 | 4.6 | NR3C1 | -14.4 | 2.7 | KMT2D | -5.2 | 2.2 |
| SS18 | -4.9 | 4.5 | CBX2 | -3.9 | 2.7 | BRD9 | -3.4 | 2.2 |
| LMNB1 | -2.2 | 4.5 | STAT1 | -2.9 | 2.7 | CREBBP | -2.3 | 2.2 |
| LEO1 | -2.5 | 4.4 | MAU2 | -15.1 | 2.6 | JARID2 | -19.0 | 2.2 |
| EBF3 | -2.1 | 4.2 | RXRA | -7.0 | 2.6 | VDR | -5.4 | 2.2 |
| STAT3 | -3.0 | 4.0 | EZH2 | -16.8 | 2.6 | XPO1 | -2.4 | 2.2 |
| IRF1 | -6.5 | 4.0 | FOXA2 | -3.3 | 2.6 | TAZ | -4.0 | 2.2 |
| FOXC1 | -3.1 | 3.8 | MEF2A | -2.5 | 2.5 | Epitope tags | -6.6 | 2.2 |
| DDIT3 | -2.3 | 3.8 | TP53 | -3.5 | 2.5 | NFE2L2 | -3.0 | 2.2 |
| GFP | -2.3 | 3.7 | EP300 | -8.2 | 2.5 | TP53BP1 | -2.5 | 2.2 |
| SUZ12 | -7.3 | 3.7 | AFF4 | -2.2 | 2.5 | CEBPB | -2.8 | 2.1 |
| TEAD4 | -4.3 | 3.7 | BCOR | -11.7 | 2.5 | CHD7 | -7.2 | 2.1 |
| BRD1 | -2.4 | 3.6 | YAP1 | -9.0 | 2.4 | SMC3 | -2.3 | 2.1 |
| STAT2 | -6.6 | 3.5 | IRF8 | -3.1 | 2.4 | TP63 | -2.4 | 2.1 |
| FLI1 | -2.1 | 3.5 | RAD21 | -2.5 | 2.4 | KDM2B | -15.7 | 2.1 |
| IRF9 | -3.9 | 3.5 | CBX7 | -10.0 | 2.4 | SMC1A | -3.0 | 2.1 |
| FOS | -12.1 | 3.4 | TLE3 | -4.2 | 2.4 | INO80 | -3.5 | 2.1 |
| ARID1A | -7.5 | 3.4 | VGLL1 | -2.1 | 2.4 | FOXA1 | -3.6 | 2.0 |
| FOXO1 | -2.4 | 3.4 | SMARCC1 | -13.0 | 2.4 | HIC1 | -2.6 | 2.0 |
| ARID2 | -2.9 | 2.0 | RUNX1 | -2.2 | 2.0 | KDM6A | -7.0 | 2.0 |
| GATA2 | -3.4 | 2.0 | TCF21 | -4.8 | 2.0 |  |  |  |

Note: FE represents “Fold Enrichment”. Feature represents “TF”.

Table S3 TFs (*p* ≤ 0.01, FE ≥ 2) with enriched binding motifs in promoters of the 361 consistent DEGs, as predicted by HOMER

| TFs | P-value | FE | TFs | P-value | FE |
| --- | --- | --- | --- | --- | --- |
| HMX1 | 1.00E-11 | 32.4 | ZSCAN21 | 1.00E-09 | 2.9 |
| TFDP1 | 1.00E-11 | 3.4 | GMEB2 | 1.00E-08 | 3.4 |
| ZFP42 | 1.00E-11 | 16.5 | POL013.1_MED-1 | 1.00E-08 | 10.0 |
| ZNF768 | 1.00E-11 | 2.3 | PRDM1 | 1.00E-08 | 2.9 |
| CEBP:AP1 | 1.00E-10 | 58.3 | REL | 1.00E-08 | 2.7 |
| FOXN1 | 1.00E-10 | 3.0 | TCF7 | 1.00E-08 | 2.5 |
| PB0208.1_ZSCAN4_2 | 1.00E-10 | 2.3 | VEZF1 | 1.00E-08 | 3.8 |
| ZNF175 | 1.00E-10 | 6.5 | NRF1 | 1.00E-07 | 9.2 |
| HNF4G | 1.00E-09 | 2.0 | PB0002.1_ARID5A_1 | 1.00E-07 | 2.7 |
| MF0010.1_HOMEOBOX_CLASS | 1.00E-09 | 2.4 | ZNF135 | 1.00E-07 | 6.2 |
| MYOG | 1.00E-09 | 2.7 | ZNF263 | 1.00E-07 | 5.9 |
| POL009.1_DCE_S_II | 1.00E-09 | 3.7 | ZNF416 | 1.00E-07 | 4.4 |
| SMAD3 | 1.00E-09 | 4.5 | PB0081.1_TCF1_1 | 1.00E-06 | 3.2 |
| ZFP335 | 1.00E-09 | 3.7 | MZF1 | 1.00E-05 | 2.2 |
| ZNF519 | 1.00E-09 | 2.3 | NR1H2 | 1.00E-05 | 3.3 |
| TEAD | 1.00E-09 | 3.5 | ZFP961 | 1.00E-06 | 117.0 |

Note: FE represents “Fold Enrichment”, and is computed as the ratio of its occurrence frequency in the target promoter set to that in the background promoter set.

Table S4 Consistent TF activities inferred by VIPER from all DEGs (log₂FC input).

| TF | X1 | X2 | X3 | X4 | X5 | X6 | X7 | Direction |
| --- | --- | --- | --- | --- | --- | --- | --- | --- |
| ATF2 | 1.0 | 2.8 | 3.2 | 4.2 | 2.1 | 4.3 | 0.6 | Consistent_Activated |
| ATF6 | 1.1 | 2.4 | 2.4 | 2.3 | 1.1 | 4.9 | 1.4 | Consistent_Activated |
| BACH1 | 2.1 | 3.0 | 3.8 | 2.9 | 0.8 | 3.5 | 2.6 | Consistent_Activated |
| CTCF | 1.7 | 2.7 | 2.0 | 1.5 | 0.9 | 1.4 | 1.0 | Consistent_Activated |
| EGR1 | 1.4 | 3.5 | 2.8 | 3.4 | 0.1 | 0.9 | 0.6 | Consistent_Activated |
| ELK1 | 0.7 | 2.0 | 3.9 | 3.3 | 1.4 | 1.6 | 1.1 | Consistent_Activated |
| ESR2 | 2.1 | 0.7 | 1.9 | 2.1 | 0.9 | 1.4 | 1.5 | Consistent_Activated |
| ETS1 | 2.9 | 2.4 | 5.0 | 4.0 | 3.5 | 5.1 | 2.1 | Consistent_Activated |
| ETV4 | 3.7 | 3.8 | 3.1 | 3.1 | 3.1 | 3.0 | 2.0 | Consistent_Activated |
| FOS | 1.4 | 2.1 | 1.3 | 0.5 | 1.6 | 2.6 | 0.5 | Consistent_Activated |
| FOSL1 | 2.9 | 4.6 | 3.4 | 4.4 | 2.3 | 3.9 | 3.2 | Consistent_Activated |
| FOSL2 | 2.2 | 3.7 | 2.6 | 2.6 | 2.0 | 1.0 | 2.5 | Consistent_Activated |
| FOXO3 | 3.1 | 3.4 | 3.5 | 4.9 | 2.1 | 2.9 | 3.0 | Consistent_Activated |
| GATA2 | 2.0 | 1.0 | 0.8 | 0.9 | 1.8 | 1.3 | 0.4 | Consistent_Activated |
| GLI2 | 1.7 | 1.5 | 1.6 | 0.5 | 1.3 | 1.9 | 1.3 | Consistent_Activated |
| HIF1A | 2.5 | 4.1 | 4.2 | 7.3 | 2.0 | 4.7 | 3.9 | Consistent_Activated |
| IRF1 | 4.9 | 5.3 | 3.7 | 2.7 | 4.3 | 4.3 | 4.7 | Consistent_Activated |
| IRF9 | 2.7 | 3.1 | 2.0 | 1.1 | 2.7 | 3.2 | 3.4 | Consistent_Activated |
| JUN | 5.4 | 8.2 | 7.7 | 7.4 | 5.5 | 6.2 | 2.7 | Consistent_Activated |
| JUND | 3.7 | 4.6 | 4.1 | 3.9 | 2.9 | 3.7 | 2.6 | Consistent_Activated |
| MYB | 0.9 | 1.6 | 1.3 | 1.1 | 0.3 | 0.6 | 2.0 | Consistent_Activated |
| NFE2L2 | 1.2 | 1.7 | 1.3 | 2.9 | 0.7 | 3.8 | 3.2 | Consistent_Activated |
| NFKB1 | 5.5 | 6.3 | 4.9 | 5.1 | 4.7 | 5.2 | 2.5 | Consistent_Activated |
| NFKB2 | 0.9 | 1.0 | 3.5 | 3.1 | 1.9 | 3.6 | 1.5 | Consistent_Activated |
| NR3C1 | 2.5 | 2.2 | 1.0 | 0.7 | 0.9 | 0.2 | 0.3 | Consistent_Activated |
| PPARA | 2.2 | 3.1 | 3.4 | 2.3 | 1.9 | 1.6 | 2.7 | Consistent_Activated |
| PPARG | 0.4 | 1.7 | 1.3 | 2.8 | 1.4 | 2.1 | 0.2 | Consistent_Activated |
| REL | 2.6 | 3.3 | 4.5 | 4.6 | 2.1 | 5.2 | 1.1 | Consistent_Activated |
| RELA | 2.3 | 3.4 | 5.0 | 4.1 | 0.8 | 3.6 | 1.4 | Consistent_Activated |
| RELB | 0.6 | 2.3 | 2.4 | 3.0 | 1.7 | 4.1 | 0.5 | Consistent_Activated |
| RUNX1 | 2.9 | 0.3 | 2.2 | 1.1 | 1.8 | 0.9 | 2.1 | Consistent_Activated |
| RUNX3 | 1.5 | 2.8 | 1.0 | 1.4 | 1.6 | 1.3 | 1.8 | Consistent_Activated |
| SMAD3 | 6.0 | 6.0 | 5.9 | 8.1 | 3.9 | 4.4 | 2.5 | Consistent_Activated |
| SP3 | 2.3 | 2.7 | 2.2 | 2.0 | 1.7 | 3.4 | 1.9 | Consistent_Activated |
| SPI1 | 5.1 | 6.3 | 4.1 | 5.7 | 2.6 | 4.3 | 5.0 | Consistent_Activated |
| STAT2 | 7.9 | 7.8 | 4.9 | 4.1 | 6.2 | 6.7 | 6.8 | Consistent_Activated |
| STAT3 | 5.0 | 6.4 | 3.8 | 4.4 | 3.1 | 2.2 | 0.5 | Consistent_Activated |
| STAT4 | 2.6 | 2.4 | 1.8 | 2.0 | 1.9 | 2.2 | 1.3 | Consistent_Activated |
| STAT5A | 0.4 | 0.2 | 3.5 | 0.8 | 1.2 | 1.0 | 1.3 | Consistent_Activated |
| STAT6 | 0.9 | 0.8 | 0.8 | 1.9 | 0.9 | 0.9 | 0.8 | Consistent_Activated |
| TFAP2A | 0.4 | 1.7 | 0.9 | 1.8 | 1.4 | 0.4 | 1.6 | Consistent_Activated |
| TP53 | 1.6 | 3.9 | 4.0 | 2.9 | 2.7 | 2.4 | 0.4 | Consistent_Activated |
| TWIST1 | 1.7 | 3.8 | 2.4 | 4.0 | 2.3 | 2.9 | 0.9 | Consistent_Activated |
| USF2 | 1.9 | 1.8 | 3.3 | 1.5 | 0.3 | 2.6 | 0.1 | Consistent_Activated |

Note: Values showed are the VIPER scores, representing a normalized enrichment score (NES) reflecting the extent to which a TF’s target genes (regulon) are coordinately up- or down-regulated in the input gene signature (log₂FC). Positive scores indicate activation, negative scores indicate inhibition. X1 to X7 represent GSE310645:NC_MSCs vs NC, GSE310645: SM_MSCs vs SM, GSE310645: LPM_MSCs vs LPM, GSE98147: SM_MSCs vs SM, GSE272480: NC_MSCs vs NC, GSE272480: TP_MSCs vs TP, and GSE182161: LPM_MSCs vs LPM, respectively.

**Reference**

1 Nguyen L, Motoike S, Zujur D, Yoshizawa K, Takashima Y, Uezumi A, et al. Embryonic lineage-specific ipsc-derived mesenchymal stem/stromal cells exhibit different morphologies and intrinsic functions,*.* iScience, 2026, 29(1): 114482.

2 Winston T, Song Y, Shi H, Yang J, Alsudais M, Kontaridis MI, et al. Lineage-specific mesenchymal stromal cells derived from human ipscs showed distinct patterns in transcriptomic profile and extracellular vesicle production*.* Adv Sci (Weinh), 2024: e2308975.

3 Wei Y, Wang B, Jia L, Huang W, Xiang AP, Fang C, et al. Lateral mesoderm-derived mesenchymal stem cells with robust osteochondrogenic potential and hematopoiesis-supporting ability*.* Front Mol Biosci, 2022, 9: 767536.

4 Nakajima T, Shibata M, Nishio M, Nagata S, Alev C, Sakurai H, et al. Modeling human somite development and fibrodysplasia ossificans progressiva with induced pluripotent stem cells*.* Development, 2018, 145(16).
